# *MUC4* mutations promote a thrombotic phenotype in patients with paroxysmal nocturnal haemoglobinuria by increasing the deposition of terminal complement

**DOI:** 10.1002/ctm2.70567

**Published:** 2026-01-05

**Authors:** Yingying Chen, Mengting Che, Chaomeng Wang, Qiaoyi Bronte Zhang, Weixin Chen, Hui Liu, Chunyan Liu, Guang Sheng Ling, Rong Fu

**Affiliations:** ^1^ Department of Hematology Tianjin Medical University General Hospital Tianjin China; ^2^ Tianjin Key Laboratory of Bone Marrow Failure and Malignant Hemopoietic Clone Control Tianjin China; ^3^ Tianjin Institute of Hematology Tianjin China; ^4^ School of Biomedical Sciences LKS Faculty of Medicine The University of Hong Kong Hong Kong China; ^5^ Division of Rheumatology and Clinical Immunology Department of Medicine Queen Mary Hospital University of Hong Kong Hong Kong China; ^6^ Department of Medicine School of Clinical Medicine LKS Faculty of Medicine The University of Hong Kong Hong Kong China

**Keywords:** C5b‐9, MUC4, paroxysmal nocturnal haemoglobinuria, thrombosis

## Abstract

**Background:**

Thrombosis is a common complication in paroxysmal nocturnal haemoglobinuria (PNH) patients, but primary prevention remains controversial. Identifying high‐risk individuals could enable risk‐stratified prophylactic anticoagulation strategies.

**Methods:**

We analyzed clinical data from PNH patients with or without thrombosis, including *MUC4* mutation status and serum complement C5b‐9 levels. Complement deposition assays and a murine lower limb deep vein thrombosis model were used to investigate the role of *MUC4* mutation in thrombotic risk and explore the underlying mechanism involving terminal complement activation in PNH patients. Therapeutic interventions with low molecular weight heparin (LMWH) were tested in vivo.

**Results:**

We found that PNH patients with *MUC4* mutations have a higher incidence of thrombotic events (TEs) and *MUC4* mutation is an independent risk factor for TE in PNH patients. Additionally, PNH patients with acute thrombosis had elevated serum complement C5b‐9 levels, and complement deposition experiments further confirmed the abnormal activation and excessive deposition of C5b‐9 as the basis for the thrombotic tendency in PNH patients. By constructing a mouse model of lower limb deep vein thrombosis, we confirmed the thrombotic tendency in a PNH mouse model and that MUC4 deficiency further promoted the thrombotic phenotype of the mice. Moreover, we found that MUC4 knockdown promoted the deposition of C5b‐9 on the cell surface, indicating that a lack of MUC4 expression facilitates the deposition of C5b‐9. Finally, in vivo drug administration experiments demonstrated that prophylactic anticoagulation with LMWH significantly reduced both the incidence of thrombosis and thrombus length in murine models.

**Conclusion:**

*MUC4* mutations promote the thrombotic phenotype in PNH patients by increasing the deposition of terminal complement. In PNH patients with concomitant MUC4 mutations, the risk of TEs is further elevated. The potential role of early complement inhibitor therapy in reducing this heightened thrombotic risk, as well as the value of prophylactic LMWH therapy as a potential option for patients who are unable to receive complement inhibitor treatment, warrants further study and prospective validation.

**Key points:**

- *MUC4* gene mutation increases the deposition of abnormally activated terminal complement in patients with PNH, thereby promoting the thrombotic phenotype in these patients. Consequently, the risk of thrombosis is further elevated in PNH patients with concurrent *MUC4* mutations.
- In patients with PNH who have concurrent *MUC4* mutations, the potential role of early complement inhibitor therapy in reducing thrombosis risk, as well as the value of prophylactic LMWH therapy as a potential alternative for those who are unable to receive complement inhibitor treatment, may warrant further study and prospective validation.

## Graphical abstract

1. PNH patients lack the complement regulatory proteins CD55 and CD59, triggering excessive activation of the terminal complement pathway and a prothrombotic state.

2. *MUC4* mutation is an independent thrombotic risk factor in PNH.

3. Mutation/down‐regulation of MUC4 promotes the abnormal deposition of excessively activated terminal complement, exacerbating a severe thrombotic phenotype.

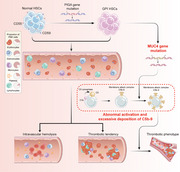

## 1 INTRODUCTION

PNH is a haematopoietic stem cell disorder caused by acquired *PIG‐A* mutations.^1^ TEs are major complications that significantly reduce survival^2^ and are the leading cause of mortality.^3^, ^4^ The increased thrombotic risk in PNH is driven primarily by complement activation,^5^, ^6^ with additional contributions from platelet activation, free haemoglobin toxicity, complement‐mediated procoagulant mechanisms and racial/genetic background.^7^, ^8^, ^9^, ^10^ Owing to the uncertain efficacy and safety of prophylactic anticoagulation, the primary method for the prevention of thrombosis in PNH patients remains controversial, highlighting the need for reliable risk stratification.

Secondary mutations contribute to TEs in PNH.^11^, ^12^ The *MUC4* gene (chr3q29) encodes a membrane‐bound mucin glycoprotein essential for cell adhesion and extracellular matrix interactions.^13^, ^14^ Our prior studies revealed *MUC4* mutations in thrombotic PNH patients,^15^, ^16^ indicating its role in thrombosis. This study further elucidated the role and mechanism of MUC4 in thrombus formation in PNH patients, providing a rational and theoretical basis for the primary prevention of thrombosis in PNH patients with concomitant *MUC4* gene mutations.

## 2 MATERIALS AND METHODS

### 2.1 Patients and clinical samples

We enrolled classic PNH patients from the Department of Hematology, Tianjin Medical University General Hospital, between January 2022 and May 2024. All patients were diagnosed following the diagnostic criteria established by the International PNH Study Group.^17^ For *MUC4* mutation analysis, whole‐exome sequencing (WES)/target region sequence (TR) data were collected from 25 patients with classic PNH,^15^, ^16^ none of whom had received prior complement inhibitor therapy. Additionally, WES data from 12 non‐PNH patients with cerebral infarction or lower extremity deep vein thrombosis (DVT) and 12 healthy controls were included for joint analysis (Table S1). Patients were followed for TEs (≤6‐month intervals) from diagnosis until February 2025 or death (median follow‐up: 5 years; range: 6 months–31 years). Notably, all patients initiated LMWH anticoagulant therapy immediately after confirmation of acute TEs. Among the PNH patients with no history of TEs, none had received anticoagulant therapy for PNH or other conditions. Two patients had been taking aspirin long‐term for coronary heart disease. Serum samples from PNH patients receiving anti‐C5 monoclonal antibody treatment (as described in Section 3.3) were obtained from a clinical trial of a C5 complement inhibitor conducted at our centre.

### 2.2 DNA sequencing

DNA from clinical samples was extracted (DP304; Tiangen), and a library was constructed by WES or TR. The Illumina platform was used for sequencing (Novogene Co., Ltd., Beijing, China), and the mutation sites were filtered using international filtering standards and previous high‐level filtering criteria used in our centre's research on mutation sites.^15^, ^16^

### 2.3 Cell lines and mice

We used previously constructed PNH cell models^18^ and PNH mouse models.^19^ Specifically, the PNH cell model refers to the monoclonal expanded PIG‐A knockout K562 cell line (K562 KO), which was generated via CRISPR–Cas9; its corresponding normal control is the wild‐type (WT) K562 cell line (K562 WT). For the PNH mouse model, haematopoietic system‐specific PIG‐A knockout mice were constructed using ES targeting technology combined with Vav‐iCre. The expression of GPI‐AP in K562 KO cells and peripheral blood (PB) cells from PNH model mice (Pig‐a[Flox/Y, Vav‐iCre]/Pig‐a[Flox/Flox, Vav‐iCre]) was completely absent (Figure S1). Using CRISPR/Cas9, we generated *Muc4* whole‐genome knockout mice (Muc4[−/−) in which exon 2 was targeted (Figure S2A). Successful knockout of Muc4 was verified by PCR and DNA sequencing (Figure S2B,C). These mice were crossed with PNH mice to generate haematopoietic tissue‐specific *Pig‐a* and *Muc4* double knockout mice (Muc4[−/−], Piga[flox/flox], Vav‐icre/Muc4[−/−], Piga[flox/Y], Vav‐icre) (Figure S2D). The expression levels of Muc4 and Piga in mouse bone marrow cells were validated by western blotting (Figure S2E).

### 2.4 Reverse transcription‑quantitative polymerase chain reaction

Leukocytes from PNH patients were isolated using erythrolysin solution (R1010; Solarbio). Total RNA was extracted (TaKaRa Bio, Inc.) and reverse transcribed (KR116; Tiangen). SuperReal PreMix Plus (SYBR Green) (FP209; Tiangen) and a Light Cycler 1.5 Real‐Time PCR system (Roche Diagnostics, Indianapolis, USA) were used for reverse transcription‑quantitative polymerase chain reaction. The specific primers used for the amplification of the target genes are listed in Table S2.

### 2.5 Western blot

Total protein was extracted using RIPA buffer (R0010; Solarbio) and separated by 4–20% SurePAGE. After the proteins were transferred to PVDF membranes, the blots were incubated overnight with anti‐MUC4 (sc‐33654; Santa), anti‐PIGA (bs‐9524R; Bioss), anti‐C5b‐9 (sc‐66190; Santa), anti‐β‐actin (4970; CST) and anti‐GAPDH (TA‐08; ZSGB‐BIO) antibodies, followed by HRP‐conjugated secondary antibodies (7074/7076; CST). Signals were detected using ECL reagent (12630; CST) and a GelView6000Plus Smart Chemiluminescence Imaging System (Guangzhou Biolight Biotechnology Co., Ltd.). ImageJ (v2.3.0) was used for densitometric analysis of the bands.

### 2.6 Construction of the thrombosis mouse model

Lower limb DVT mouse models were established in four groups (*n* = 10; 1:1 M:F): normal C57BL/6 mice (WT), Muc4 whole‐genome knockout mice (Muc4‐KO), haematopoietic Piga gene knockout mice (Piga‐KO) and haematopoietic Piga and Muc4 double‐knockout mice (Piga/Muc4‐DKO) (15‐week‐old). Prior to surgical or pharmacological interventions, all the mice were acclimatised in individually ventilated cages (IVCs) for 1 week. After anaesthesia, an incision was made along the midline of the abdomen, and a 7–0 suture was used to completely ligate the left iliac vein at the proximal end. Afterward, the incision was sutured layer by layer (Figure S3A). A sham operation group and an untreated group were established. Twenty‐four hours after surgery, the mice were euthanised, and PB and local thrombus tissues were collected and processed for subsequent experimental analyses.

We carefully dissected vascular and thrombus tissues. The tissues were either lysed in RIPA buffer and homogenised for Western blot (WB) analysis or fixed in 4% paraformaldehyde. After fixation, the tissues were photographed, and thrombus lengths were measured using a stereo microscope. The fixed thrombus tissues were then embedded in paraffin, sectioned and baked. Finally, the sections were stained with haematoxylin and eosin (H&E) and observed under a microscope to analyse the tissue structure.

### 2.7 Enzyme‐linked immunosorbent assay

Human and mouse serum C5b‐9 levels were quantified using species‐specific enzyme‐linked immunosorbent assay (ELISA) kits (SBJ‐H1726 and SBJ‐M0335; SenBeiJia) following the manufacturer's protocols. A standard curve was generated according to the standard product concentration, and the sample pore concentration was calculated according to the standard curve.

### 2.8 Immunohistochemical analysis

C5b‐9 deposition in the local thrombus tissue was detected by immunohistochemical (IHC) analysis. After deparaffinisation and antigen retrieval (citrate buffer/EDTA buffer), the sections were treated with 3% H_2_O_2_, blocked with goat serum (C0265; Beyotime) and incubated with an anti‐C5b‐9 antibody (bs‐2673R; Bioss) overnight. Secondary antibodies (SSA004; Sino Biological) and DAB (ZLI‐9018; ZSGB‐BIO) were used for detection, and the sections were counterstained with haematoxylin. Slides were scanned by a NanoZoomer S210 (Hamamatsu, Japan) and analysed by SlideViewer software 2.6.

### 2.9 Flow cytometry and complement deposition experiments

Flow cytometry was performed to analyse C5b‐9 deposition on cell surfaces.^20^ K562 WT and K562 KO cells were seeded in 96‐well plates in PBS or in Gelatin Veronal Buffer with Mg^2+^ and EGTA (GVBMG, pH 6.4) (BR4003603; Bioleaper) to activate the alternative complement pathway or in Gelatin Veronal Buffer (GVB^2+^, pH 7.4) (G6514; Sigma‒Aldrich) to activate all complement pathways. After 20 µL of PNH patient serum or normal control serum was added and incubated at 37°C for 15 min, the reactions were terminated by the addition of 1% bovine serum albumin and EDTA. The cells were subsequently washed and stained with an anti‐C5b‐9 monoclonal primary antibody (sc‐58935; Santa) and an APC‐conjugated secondary antibody (bs‐0296G‐APC; Bioss) for 30 min. Normal human serum containing EDTA was used as the negative control, and C5b‐9 deposition is expressed as the percentage of positive cells relative to the parent population. The samples were assessed with a Beckman Coulter system, and the results were analysed using CytExpert software and FlowJo 10.9.0.

### 2.10 siRNA transfection

MUC4‐targeting siRNAs (Beijing MIJIA Technology Co., Ltd.; sequences in Table S3) were transfected into K562 KO cells using Lipofectamine RNAiMAX (13778150; Thermo Fisher Scientific) according to the manufacturer's protocol. Briefly, siRNA/RNAiMAX complexes were prepared in Opti‐MEM (31985070; Thermo Fisher Scientific), incubated for 20 min at room temperature and then added to six‐well plates containing K562 KO cells. The transfected cells were harvested 48 h after transfection.

### 2.11 Prophylactic anticoagulation with LMWH

Mice (15‐week‐old) received prophylactic LMWH (Clexane, Sanofi Winthrop Industrie; 100 or 300 IU/kg/d) or PBS control via subcutaneous injection for 7 days. Blood parameters and bleeding/clotting times (BT and CT) were assessed posttreatment. On day 8, DVT models were established, with thrombus collection occurring on day 9 posteuthanasia.

### 2.12 Determination of mouse BT and CT

Following anaesthesia, the mouse tail tip was cut 0.5 cm from the tip. The distal 5 cm of the tail was then immersed in 37°C saline, and BT was measured as the duration of active bleeding observed in the saline solution. For CT, orbital blood was placed on a glass slide and monitored every 30 s until fibrin threads appeared upon gentle stirring with a blunt needle. The time from the start of blood collection to the observation of the blood threads was defined as the CT.

### 2.13 Statistical analysis

Statistical analyses were conducted using SPSS 26.0 and GraphPad Prism 8.2.1. Continuous data are expressed as the mean ± standard deviation, while categorical variables are expressed as numbers (percentages). Group comparisons utilised *t*‐tests/Mann‒Whitney tests (two groups) or chi‒square/Kruskal‒Wallis tests (multiple groups). Multivariate TE risk factors were assessed by logistic regression. Spearman's correlation was used to evaluate the correlation between quantitative variables. A *p* value < .05 was considered to indicate statistical significance.

## 3 RESULTS

### 3.1 *MUC4* gene mutations are closely related to TEs in PNH patients

In our previous studies, we reported that some PNH patients with thrombosis had *MUC4* mutations. To clarify the relationship between *MUC4* mutations and TEs, we conducted a combined analysis of WES/TR data from 25 PNH patients (PNH group), 12 non‐PNH patients with acute TEs (TE group) and 12 healthy controls (HC group) (Supporting Information 2). In the PNH group, 12 patients (PNH‐TE subgroup) developed TEs following PNH diagnosis, whereas the remaining 13 PNH patients (PNH non‐TE subgroup) did not experience any TEs. The TE incidence was 48% (12 out of 25), with a median time from diagnosis to TE occurrence of 3 years (range: 0 days to 15 years). Notably, five patients experienced secondary TEs. Cerebral thrombosis (5) and lower extremity DVT (5) were the most frequently observed TEs, with other embolic sites including splenic infarction (2), mesenteric vein thrombosis (2), portal vein thrombosis (1), pulmonary embolism (1) and myocardial infarction (1). No *PIG‐A* mutations were detected in the TE or HC group. In the PNH group, 11 patients (44%) had *PIG‐A* mutations. The *PIG‐A* mutation rates in the PNH‐TE and PNH non‐TE groups were 50.0% (six out of 12) and 38.5% (five out of 13), respectively, with no significant difference (*p *= .430).

**FIGURE 1:**
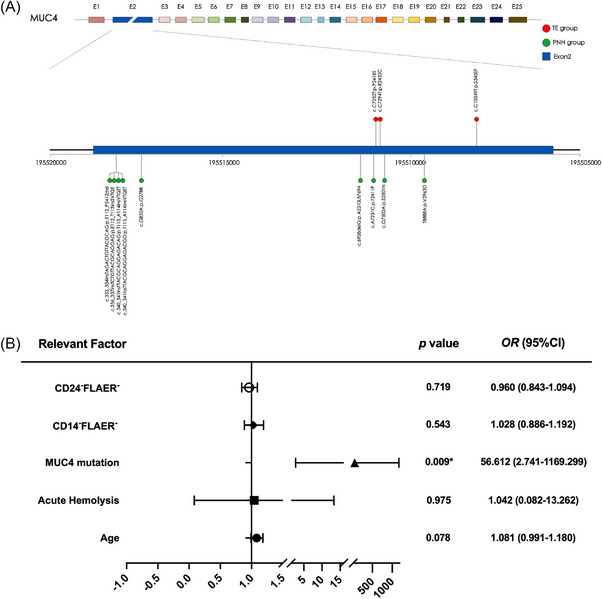
*MUC4* mutation is closely related to TEs in PNH patients. (A) Schematic diagram of the *MUC4* mutation sites detected in the PNH group (green) and the TE group (red). (B) Forest plot showing the logistic regression results of factors associated with TEs in PNH patients.

We analysed the *MUC4* mutation sites that were unique to the PNH and TE groups after comparison with those in the HC group. The incidence of *MUC4* gene mutations was significantly greater in PNH patients (nine out of 25, 36%) than in TE patients (two out of 12, 16.7%) (Table 1 and Figure 1A) (*p *= .003). Furthermore, the *MUC4* mutation rate in the PNH‐TE subgroup was significantly greater (eight out of 12, 66.7%) than that in the PNH non‐TE subgroup (one out of 13, 7.7%) (*p *= .003), suggesting that *MUC4* mutations are closely related to TEs in PNH patients. We compared the clinical indicators between the PNH‐TE subgroup and the PNH non‐TE subgroup. Univariate analysis revealed that advanced age, elevated haemolysis indicators (total bilirubin and free haemoglobin), high d‐dimer levels, high PNH clone proportions and *MUC4* mutations were risk factors for TEs in PNH patients (Table 2). Given the sample size constraints of this study, we did not perform survival analysis. Instead, we included five factors – those with statistical significance (*p* < .05) in the univariate analysis and clear clinical relevance – in the logistic regression model. Cross‐validation was conducted to assess model performance, confirming good predictive accuracy and a low risk of overfitting. Logistic regression further verified that M*UC4* mutation (OR = 56.6; *p *= .009) was an independent risk factor for TEs in PNH patients (Figure 1B). Owing to the limited sample size, we did not perform further statistical analysis of the clinical data between MUC4 WT and mutant (MT) patients in the PNH‐TE and PNH non‐TE groups (Table S4).

**TABLE 1:** Summary of MUC4 mutation sites.

Function change	Exonic function change	Amino acid change	Patients
Exonic	Missense SNV	exon2:c.T8888A:p.V2963D	P3, P14, P18
Exonic	Missense SNV	exon2:c.G7502A:p.S2501N	P23
Exonic	Missense SNV	exon2:c.A7231C:p.T2411P	P19
Exonic	Frameshift deletion	exon2:c.6928delG:p.A2310Lfs*694	P4
Exonic	Missense SNV	exon2:c.G832A:p.G278R	P24
Exonic	Nonframeshift insertion	exon2:c.340_341insTTACGCAGGAGACGG:p.T113_A114insVTQET	P2, P9
Exonic;splicing	Nonframeshift insertion	exon2:c.340_341insTTACGCAGGAGACAG:p.T113_A114insVTQET	P2, P9
Exonic;splicing	Nonframeshift insertion	exon2:c.336_337insTCTGTTACGCAGGAG:p.E112_T113insSVTQE	P9
Exonic	Stopgain	exon2:c.333_334insTAGACTGTTACGCAG:p.E112_P5412del	P9
Exonic	Missense SNV	exon2:c.C10349T:p.S3450F	T1
Exonic	Missense SNV	exon2:c.C7294T:p.R2432C	T10
Exonic	Missense SNV	exon2:c.C7252T:p.P2418S	T10

**TABLE 2:** Univariate analysis of factors associated with TEs in PNH patients.

Clinical feature	Measurement			
	PNH‐TE (*N* = 12)	PNH non‐TE (*N* = 13)	Reference range	*p* Value
Sex, *n* (%)				.144
Male	9 (75.0)	6 (46.2)		
Female	3 (25.0)	7 (53.8)		
Median age (range) (years)	50.5 (25–68)	33 (17–67)		.040*
Blood examination				
RBC (×10^12^/L)	2.17 ± 0.37	2.15 ± 0.57	4.30–5.80	.955
WBC (×10^9^/L)	4.68 ± 1.58	4.28 ± 2.13	3.50–9.50	.599
HGB (g/L)	72.08 ± 12.59	65.62 ± 17.23	130–175	.298
PLT (×10^9^/L)	113.0 ± 76.76	99.38 ± 77.69	125–350	.664
Ret%	8.64 ± 4.24	5.87 ± 3.86	0.50–1.5	.101
LDH (U/L)	1491.0 ± 857.2	958.31 ± 606.79	120–250	.084
TBIL (µmol/L)	36.25 ± 16.93	23.28 ± 11.31	≤26	.033*
IBIL (µmol/L)	22.48 ± 11.28	16.28 ± 8.60	19–26	.135
PT (s)	12.03 ± 1.46	11.46 ± 1.03	9.5–15.0	.274
PT‐INR	1.10 ± 0.13	1.05 ± 0.09	0.80–1.50	.270
APTT (s)	26.75 ± 3.32	25.92 ± 2.87	20.0–40.0	.511
TT (s)	21.51 ± 4.26	23.52 ± 10.10	13.0–25.0	.530
FIB (g/L)	3.24 ± 1.31	2.84 ± 1.23	1.80–4.0	.441
dd‐Dimer (ng/mL)	2732 ± 2880.06	705.38 ± 559.55	0–500	.034*
FHB (mg/L)	261.08 ± 221.35	92.38 ± 51.73	0–40	.024*
Haptoglobin (g/L)	0.13 ± 0.16	0.26 ± 0.44	0.5–2	.351
CD59^−^ erythrocyte (%)	42.30 ± 26.72	39.15 ± 30.57	<1	.787
CD59^−^ granulocyte (%)	84.63 ± 8.21	71.08 ± 18.82	<1	.030*
CD14^−^FLAER^−^ (%)	84.87 ± 9.0	71.76 ± 19.03	<1	.039*
CD24^−^FLAER^−^ (%)	87.59 ± 9.98	73.11 ± 21.39	<1	.042*
*MUC4* mutation, *n* (%)				.003*
Yes	8 (66.7)	1 (7.7)		
No	4 (33.3)	12 (92.3)		
*PIG‐A* mutation, *n* (%)				.430
Yes	6 (50.0)	5 (38.5)		
No	6 (50.0)	8 (61.5)		

Abbreviations: RBC, red blood cell; WBC, white blood cell; HGB, haemoglobin; PLT, platelet; Ret: reticulocyte; HP, haptoglobin; LDH, lactic dehydrogenase; TBIL, total bilirubin; IBIL, indirect bilirubin; PT, prothrombin time; PT‐INR, prothrombin time–international normalised ratio; APTT, activated partial thromboplastin time; TT, thrombin time; FHB, free haemoglobin; HP, haptoglobin; FLAER, fluorescently labelled aerolysin) (*p < .05).

Among the nine patients with *MUC4* mutations, eight (88.9%) experienced TEs during the disease course, whereas among the 16 patients without *MUC4* mutations, only four (25.0%) experienced TEs. The incidence of TEs was significantly greater in patients with *MUC4* mutations than in those without mutations (*p *= .003). These results suggest that *MUC4* mutations are closely related to TEs and that *MUC4* mutations increase the risk of TEs in PNH patients. Notably, all *MUC4* mutations in the patients occurred in exon 2. Given the important role of the central tandem repeat domain encoded by exon 2 in MUC4‐mediated anti‐cell adhesion,^21^ we hypothesised that *MUC4* mutations in PNH patients may promote thrombosis formation by affecting cell adhesion.

### 3.2 Down‐regulation of MUC4 expression is associated with TEs in PNH patients

**FIGURE 2:**
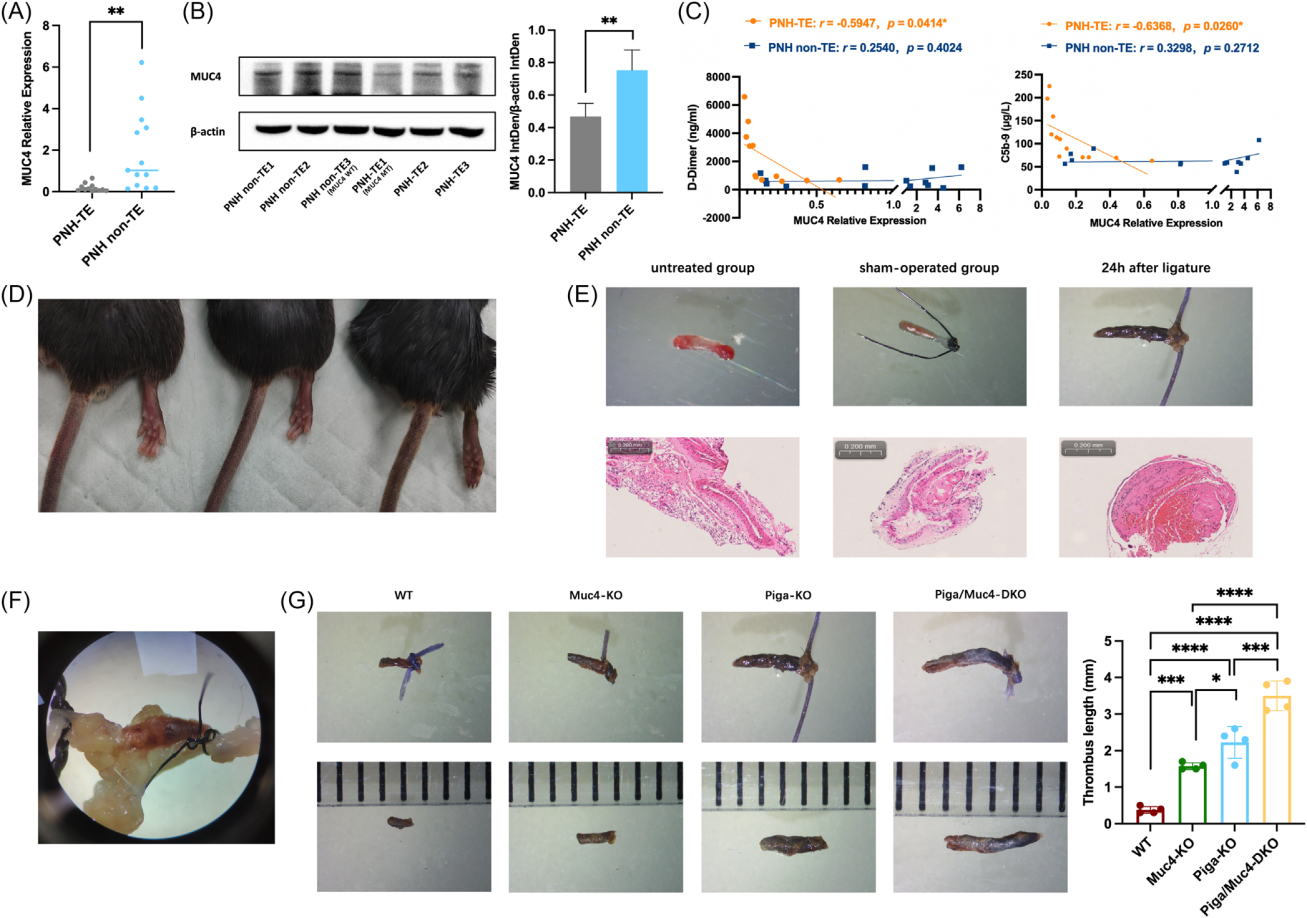
Down‐regulation of MUC4 expression promotes thrombosis. (A) qRT‒PCR detection of MUC4 mRNA expression in PB leukocytes of PNH patients with acute TEs (PNH‐TE) and PNH patients without TEs (PNH non‐TE); (B) WB detection of MUC4 protein expression in PB leukocytes of the PNH‐TE and PNH non‐TE groups; (C) correlation analysis of the MUC4 mRNA expression levels in PB leukocytes of PNH patients with plasma d‐dimer levels and serum C5b‐9 levels; (D) ligation of the unilateral iliac vein resulted in loss of motor function, cyanosis and numbness in the ipsilateral limb; (E) observation of local vascular tissue using a stereomicroscope (10×) and histological examination by H&E staining after modelling; (F) microscopic observation 24 h after modelling showed complete occlusion of the vascular lumen by thrombus (10×); (G) measurement and statistical analysis of thrombus length in four groups of mice: normal C57BL/6 mice (WT), Muc4 whole‐genome knockout mice (Muc4‐KO), haematopoietic Piga gene knockout mice (Piga‐KO) and haematopoietic Piga and Muc4 double‐knockout mice (Piga/Muc4‐DKO) at 24 h after modelling. (*p < .05, **p < .01, ***p < .001, ****p < .0001).

We further determined the expression levels of MUC4 in the PB leukocytes of PNH patients with acute TEs (PNH‐TE group) and PNH patients without thrombosis (PNH non‐TE group). The results revealed that compared with those in the PNH non‐TE group, the mRNA and protein levels of MUC4 in the PNH‐TE group were significantly lower (*p *= .0054, *p *= .0196) (Figure 2A,B). Correlation analysis revealed no correlation between MUC4 mRNA expression levels and PNH clone size or haemolysis‐related indicators (Figure S4). However, the MUC4 mRNA expression level in acute thrombotic patients was negatively correlated with the plasma dD‐dimer level (*r *= −.5947; *p *= .0414) and serum C5b‐9 level (*r *= −.6368; *p *= .0260) (Figure 2C). These results suggest that the down‐regulation of MUC4 expression is associated with the occurrence of TEs in PNH patients and that terminal complement may also be involved.

To determine the relationship between MUC4 mutation/down‐regulation and TEs, we constructed a lower limb DVT model by ligating the unilateral iliac vein (Figure S3A) and compared the incidence of thrombosis and thrombus length among the WT, Muc4‐KO, Piga‐KO and Piga/Muc4‐DKO groups after surgery. After ligation, the mice exhibited poor appetite, loss of activity in the ligated limb, limb swelling, cyanosis, rigidity and loss of sensation (Figure 2D), whereas the sham‐operated group showed no obvious abnormalities. Obvious thrombi, which were soft and loosely attached to the vessel wall, were visible 12 h after ligation, and the thrombi became firmly fixed 24 h after ligation, as shown by H&E staining (Figures 2E and S3B). At 24 h after ligation, all groups of mice developed thrombosis (Figure 2F), and the thrombus length in the Muc4‐KO and Piga‐KO groups was significantly longer than that in the WT group, while the Piga/Muc4‐DKO group had the longest thrombi (Figure 2G). These results demonstrated that mice with PNH tend to experience thrombosis and that MUC4 deficiency further promotes the thrombotic phenotype.

### 3.3 Abnormal activation and excessive deposition of terminal complement are the basis of thrombotic tendencies in PNH patients

**FIGURE 3:**
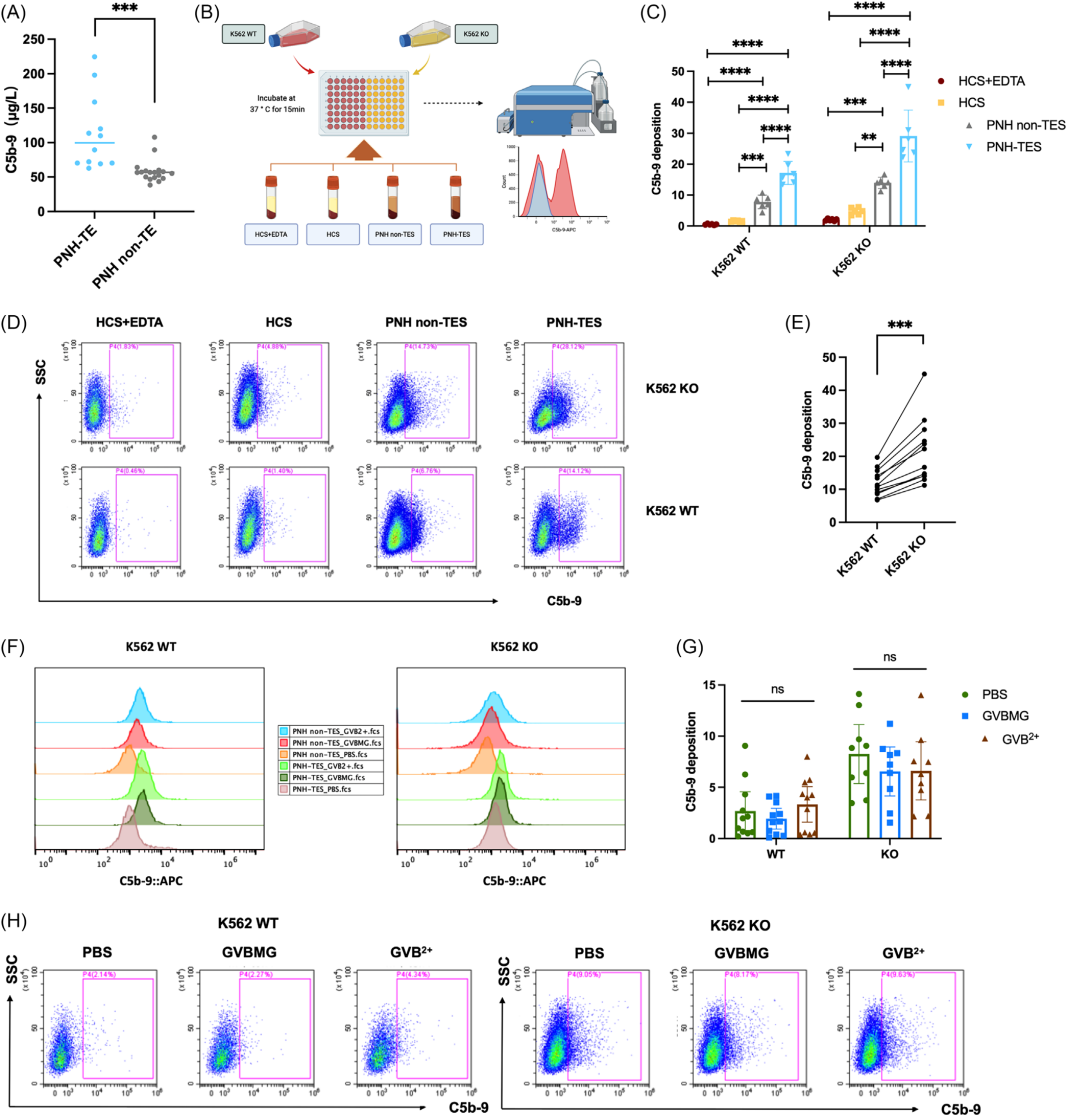
Abnormal activation and excessive deposition of terminal complement are the basis for thrombotic tendencies in PNH patients. (A) ELISA detection of serum C5b‐9 levels in the PNH‐TE and PNH non‐TE patient groups; (B) schematic diagram of the method used to detect complement C5b‐9 deposition on the surface of K562 WT and K562 KO cells using flow cytometry (created By Biorender); (C) statistical graph of complement C5b‐9 deposition levels on the surface of K562 WT and K562 KO cells after incubation with serum from different sources; (D) flow cytometric results of complement C5b‐9 deposition on the surface of K562 WT and K562 KO cells after incubation with serum from different sources; (E) comparison of complement C5b‐9 deposition levels on the surface of K562 WT and K562 KO cells after coincubation with the same patient's serum; (F) detection of changes in cell surface complement C5b‐9 deposition after activation complement with GVBMG or GVB^2+^ buffer; (G) statistical graph of complement C5b‐9 deposition on K562 WT and K562 KO cells coincubated with serum from PNH patients receiving C5 antibody treatment under conditions of no complement activation, GVBMG activation or GVB^2+^ activation; (H) flow cytometric results of complement C5b‐9 deposition on K562 WT and K562 KO cells co‐incubated with serum from PNH patients receiving C5 antibody treatment, under conditions of no complement activation, GVBMG activation or GVB^2+^ activation. (*p < .05, **p < .01, ***p < .001, ****p < .0001).

Abnormal activation of terminal complement plays a major role in TEs in PNH patients. Our data also revealed that the serum level of C5b‐9 in PNH patients with acute thrombosis was significantly greater than that in patients without thrombosis (*p *= .0003) (Figure 3A). We coincubated serum from PNH patients with thrombosis (PNH‐TES), serum from PNH patients without thrombosis (PNH non‐TES) and normal human serum (HCS) with K562 WT or K562 KO cells and detected the deposition of terminal complement C5b‐9 on the cell surface. HCS+EDTA was used as a negative control (Figure 3B). The results confirmed that compared with that in K562 cells coincubated with HCSs, the deposition of complement C5b‐9 in K562 cells coincubated with PNH patient serum was significantly greater, with the highest C5b‐9 deposition occurring in the cells coincubated with PNH‐TES (Figure 3C,D). Furthermore, after coincubation with the same patient serum, the deposition of C5b‐9 on K562 KO cells was significantly greater than that on K562‐WT cells (Figure 3E). Through a complement deposition experiment, we further confirmed that the level of serum C5b‐9 deposited on the surface of cells from PNH patients with thrombosis was increased and that abnormal PNH cells had increased terminal complement deposition, which may be related to the abnormal cell adhesion caused by the lack of GPI‐APs.

When buffer solutions were added to the coincubation system to activate the alternative complement pathway (using GVBMG) or simultaneously activate the classic pathway and alternative pathways (using GVB^2+^), the deposition of C5b‐9 on the cell surface in both groups further increased compared with that in the noncomplement activation group (PBS), especially in the K562 KO cells coincubated with PNH‐TES (Figures 3F and S5). The alternative complement pathway is recognised as the core pathogenic pathway in PNH, contributing to more than 80% of downstream complement activation products.^22^ Consistent with these findings, our results revealed comparable increases in C5b‐9 levels between the GVBMG and GVB2+ groups, suggesting that the alternative pathway is the major contributor to enhanced complement activation in PNH patients. In addition, we assessed the deposition of C5b‐9 on the cell surface after coincubation with serum from PNH patients who received anti‐C5 monoclonal antibody treatment. Although a small amount of terminal complement deposition on the cell surface was present, there was no significant increase in cell C5b‐9 deposition after complement pathway activation (Figure 3G,H), demonstrating that the anti‐C5 monoclonal antibody can effectively block the formation of C5b and inhibit the activation of the terminal complement. Although abnormal activation and excessive deposition of terminal complement make PNH patients prone to thrombosis, not all patients experience TEs during the course of the disease, indicating that there may be other factors that further promote the development of the thrombotic phenotype.

### 3.4 Loss of MUC4 expression promotes the thrombotic phenotype in PNH patients by increasing terminal complement deposition

**FIGURE 4:**
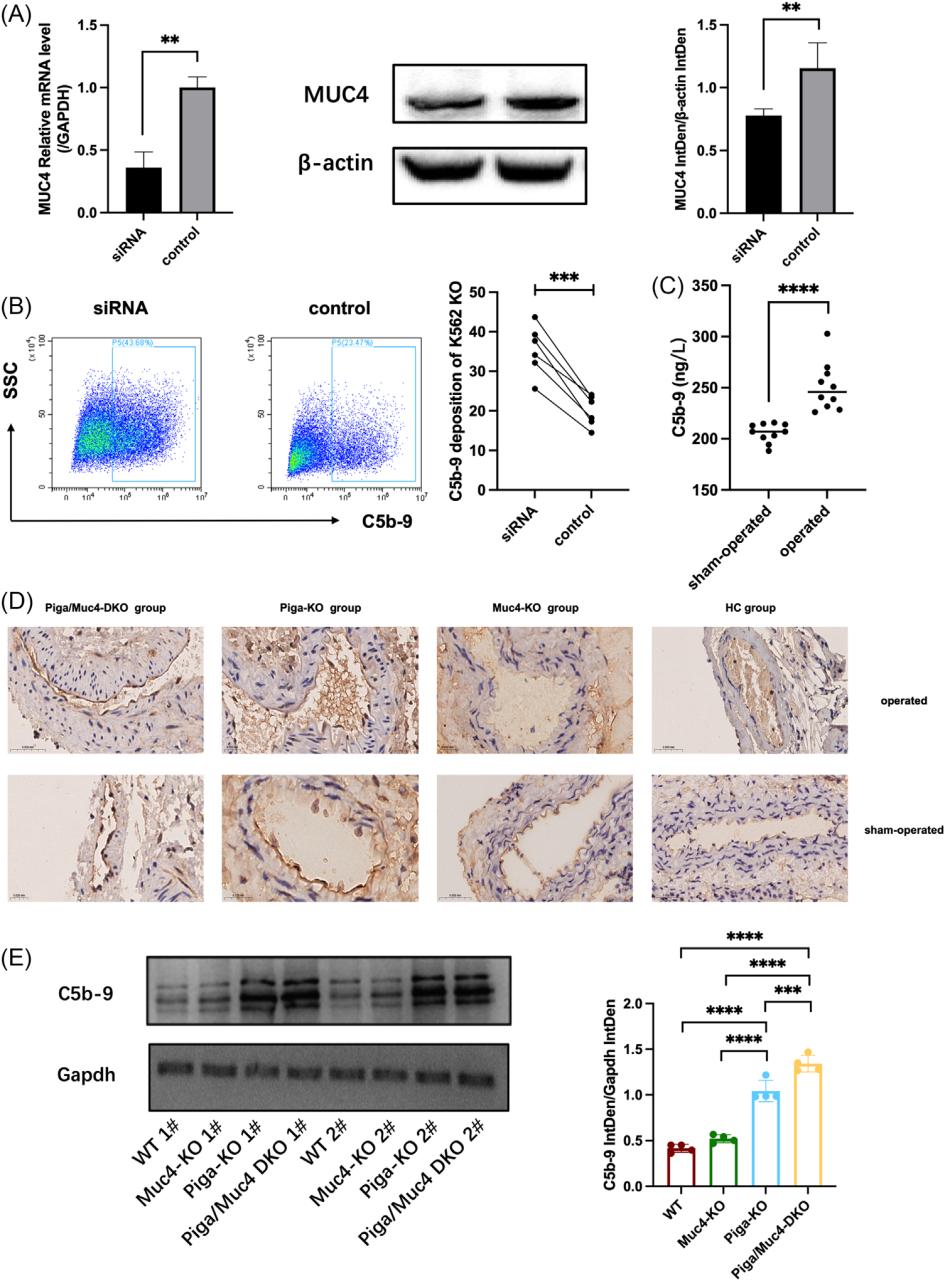
MUC4 expression promotes the development of a thrombotic phenotype in PNH patients by increasing terminal complement deposition. (A) Knockdown of MUC4 using siRNA, with verification of the knockdown efficacy by qRT‐PCR and WB analyses; (B) detection of changes in cell surface complement deposition levels after coincubation of MUC4 knockdown K562 KO cells with serum from PNH patients with acute thrombosis; (C) ELISA detection of changes in serum complement levels in the Piga/Muc4‐DKO mice; (D) IHC results of C5b‐9 in the local vascular tissue of mice; (E) WB detection of C5b‐9 protein expression in the local vascular tissue of the four groups. (*p < .05, **p < .01, ***p < .001, ****p < .0001).

Since *MUC4* mutation can increase the risk of thrombosis in PNH patients and given the important role of MUC4 in antiadhesion, we hypothesised that the loss of MUC4 expression may promote the thrombotic phenotype in PNH patients by increasing the deposition of terminal complement on the cell surface. Therefore, we used siRNA to knockdown MUC4 expression in K562 KO cells (Figure 4A) and then coincubated these cells with PNH‐TES. After MUC4 expression was knocked down, the deposition of terminal complement on the cell surface significantly increased (*p *= .0004) (Figure 4B).

We used ELISA to measure serum C5b‐9 levels in Piga/Muc4‐DKO group mice, and the results revealed that the serum C5b‐9 level in the mice with DVT was greater than that in the sham‐operated mice (Figure 4C), which may be related to further activation of complement by acute TE. We also performed IHC and WB analyses of local thrombotic tissues. The IHC results revealed ring‐like deposition of C5b‐9 along the luminal side of occluded blood vessels in Piga/Muc4‐DKO group mice. Localised C5b‐9 deposition was also observed on the luminal side of blood vessels in Piga‐KO mice, whereas no significant C5b‐9 deposition was detected in Muc4‐KO or WT mice (Figure 4D). Notably, C5b‐9 deposition was similarly present on the luminal side of local blood vessel walls in Piga/Muc4‐DKO group mice that did not undergo surgery, indicating that this terminal complement deposition was primarily caused by the disease itself rather than surgical intervention or thrombosis formation. WB analysis of local thrombotic tissues provided a more accurate quantitative assessment of local C5b‐9 deposition. Consistent with the IHC results, we observed increased C5b‐9 expression in the thrombotic vessels of Piga/Muc4‐DKO mice (Figure 4E).

Based on these findings, we propose a potential mechanism for TEs in PNH patients: *PIG‐A* mutation leads to deficiency of the complement regulatory proteins CD55 and CD59 on cell surfaces, resulting in abnormal activation of terminal complement and, consequently, thrombotic tendency. Furthermore, MUC4 mutation/down‐regulation promotes the deposition of abnormally activated terminal complement on cell surfaces, thereby increasing thrombosis risk in these patients and ultimately leading to a severe thrombotic phenotype.

**FIGURE 5:**
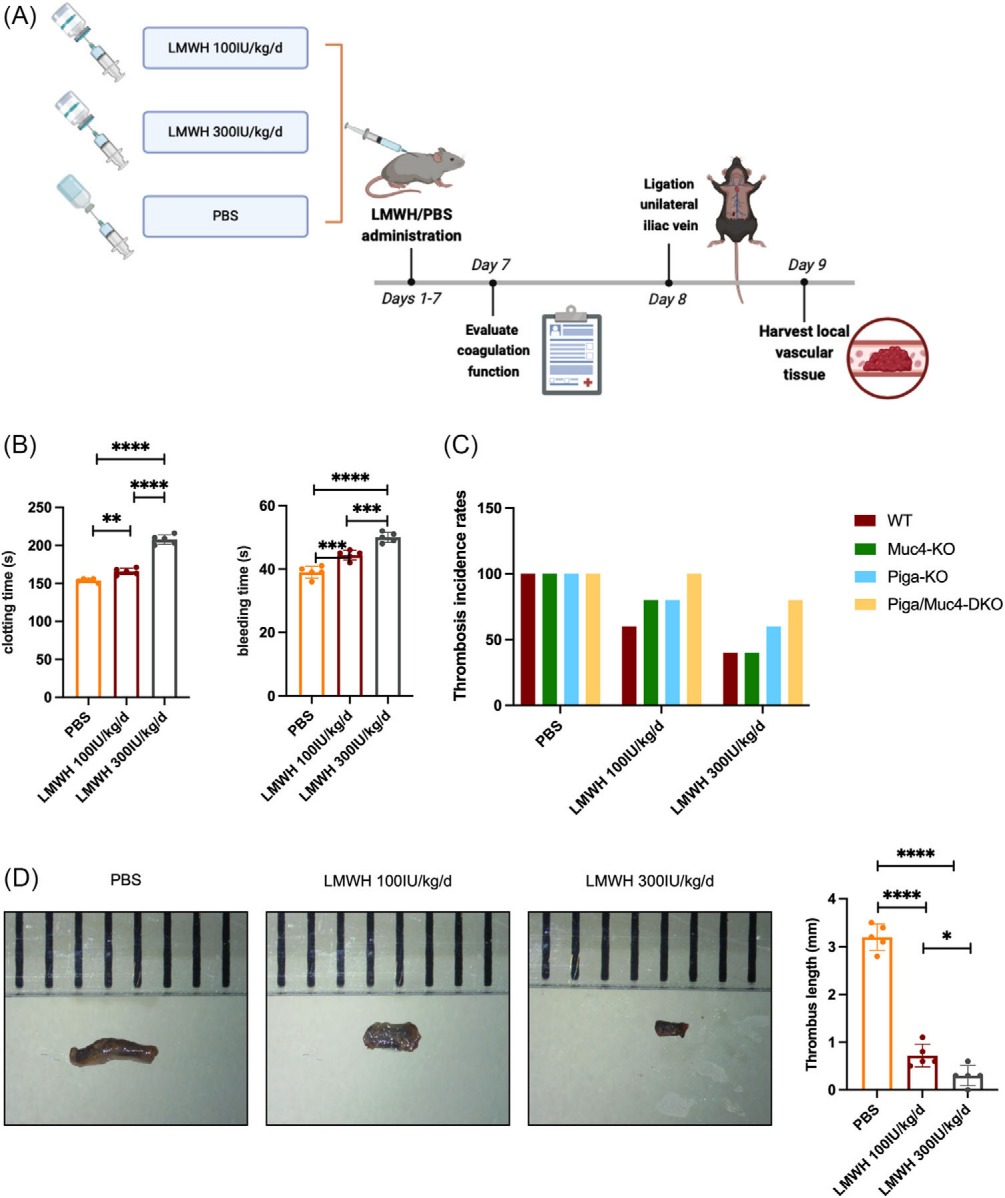
LMWH prophylactic anticoagulation is an effective and safe approach for reducing thrombotic events in PNH mice. (A) DVT modelling in mice after LMWH prophylactic anticoagulation treatment (created by Biorender); (B) detection of mouse BT and CT 7 days after LMWH prophylactic anticoagulation treatment; (C) thrombosis rates 24 h after DVT modelling; (D) measurement and statistical analysis of thrombus length 24 h after DVT modelling. (*p < .05, **p < .01, ***p < .001, ****p < .0001).

The above experiments demonstrate the crucial role of *MUC4* mutations in TEs among PNH patients. For PNH patients with *MUC4* mutations, we recommend early initiation of complement inhibition therapy to reduce thrombosis risk. However, for patients who are unable to receive complement inhibition therapy, whether prophylactic anticoagulation can effectively prevent TEs becomes a critical question. In our study, mice were administered LMWH as a prophylactic anticoagulation therapy at two doses (100 and 300 IU/kg/day) for 7 consecutive days (Figure 5A). After anticoagulation treatment, there were no significant changes in the routine blood parameters (Table S5), while the BT and CT results were prolonged (Figure 5B) without acute haemorrhage events. On day 8, unilateral iliac vein ligation was performed, and LMWH treatment reduced the thrombosis incidence in the mice (Figure 5C). In Piga/Muc4‐DKO mice, both the nonprophylactic anticoagulation group and the LMWH 100 IU/kg/day group demonstrated thrombosis rates of 100%, whereas the LMWH 300 IU/kg/day group had an 80% (four out of five) thrombosis rate. Notably, prophylactic LMWH significantly shortened the thrombus length across all groups (Figures 5D and S6). These murine data suggest that prophylactic LMWH therapy may represent a potential therapeutic option for reducing TEs in *MUC4*‐MT PNH patients who are not receiving complement inhibition therapy. Even patients with thrombocytopenia or full‐dose anticoagulation intolerance might benefit from reduced‐dose LMWH. Based on these findings, we plan to implement prophylactic anticoagulation strategies for this specific patient population in clinical practice, with long‐term follow‐up to monitor the occurrence of TEs and obtain more robust evidence.

## 4 DISCUSSION

The *MUC4* gene encodes the MUC4 protein, a membrane‐bound mucin whose rat homolog is the sialomucin complex (SMC),^23^ which masks the cell surface and spatially hinders cell‒cell, cell‒extracellular matrix and cell‒molecule interactions, exerting antiadhesive effect.^24^ Multiple studies have shown that abnormal cell adhesion after MUC4/SMC overexpression leads to cell detachment, cell‒cell separation^25^ and weakened binding between cells and extracellular matrix components.^26^ In this study, we confirmed that *MUC4* mutation is an independent risk factor for thrombosis in PNH patients. Although the *MUC4* mutation site is different in PNH patients, resulting in different amino acid changes, all mutations occur in exon 2, which encodes the tandem repeat domain. Given the important role of the tandem repeat domain in the antiadhesive function of cells^21^ and the crucial role of cell adhesion in thrombus formation in PNH patients,^16^ we speculate that *MUC4* gene mutation promotes thrombosis in PNH patients by affecting cell adhesion.

Our results revealed reduced MUC4 expression in acute TE patients (negatively correlated with d‐dimer and C5b‐9) and higher d‐dimer levels in MUC4‐MT patients than in WT patients (3217 vs. 689 ng/mL). d‐Dimer levels increase during active thrombosis and fibrinolysis, and increased procoagulant activity promotes thrombin/fibrin formation, resulting in higher D‐dimer levels in PNH patients.^27^ Some scholars suggest that dD‐dimer is a useful marker of haemostatic activation/thrombosis risk in PNH patients, suggesting that routine follow‐up of d‐dimer levels should be performed;^28^ for patients who are not receiving complement inhibitors, persistent elevation of d‐dimer levels may warrant the consideration of prophylactic anticoagulation.^29^ These results further link MUC4 mutation/absence to the occurrence of acute TEs in PNH patients. Given the limited sample size of PNH patients with acute thrombosis in this study and d‐dimer susceptibility to nonthrombotic factors (e.g., infection), single dD‐dimer measurements have limited clinical reference value. Thus, in future studies, we will further increase the sample size to increase the reliability of the results and conduct dynamic D‐dimer monitoring, combining imaging examinations and clinical symptoms, to clarify the dynamic association between MUC4 mutation/absence and thrombosis risk, thereby enhancing its clinical guiding value.

The pathogenesis of TE in PNH involves multiple synergistic factors: complement activation, endothelial dysfunction, haemolysis, reduced nitric oxide bioavailability, platelet/neutrophil activation and a vicious cycle involving the coagulation cascade, complement system and inflammatory cytokines.^2^, ^3^, ^7^, ^29^, ^30^ Among these, the complement system is the main driver of PNH thrombosis,^2^, ^31^ which is supported by a reduced TE incidence in PNH patients after eculizumab treatment.^30^, ^32^ Specifically, PNH platelets and red blood cells, which lack regulatory factors such as CD55/CD59, are vulnerable to complement attack. C5b‐9 (MAC) deposition triggers platelet activation, aggregation, proinflammatory factor release,^3^ and intravascular haemolysis.^33^ Crucially, the formation of MACs (not upstream complement products) induces PNH thrombosis.^34^ MAC‐mediated haemolysis releases ADP, activating platelet and endothelial prothrombotic activity.^35^ Moreover, complement products induce proinflammatory cytokines,^36^ regulate coagulation factor expression/activity^37^ and activate factors such as prothrombin;^38^ thrombin in turn activates complement, resulting in the formation of a “thrombus‐complement” cascade^.^ ^7^ Additionally, C5a induces neutrophil tissue factors expression,^39^ and monocyte extracellular vesicles further promote coagulation.^40^ PNH also has fibrinolytic defects, possibly linked to the deficiency of fibrinolysis‐related GPI/GPI‐APs ^41^, ^42^ and the release of fibrinolysis‐inhibiting substances by activated platelets/neutrophils^.^ ^43^ Complement and coagulation cascades cooperate, ultimately causing pathological thrombosis in PNH patients.^7^ Our study revealed elevated serum C5b‐9 levels in PNH patients with acute TEs. Complement deposition experiments further confirmed that C5b‐9 deposition on the cell surface also increased, and this phenomenon became more obvious after complement activation. Furthermore, after coincubation with serum from the same patient, compared with K562 WT cells, K562 KO cells presented significantly greater C5b‐9 deposition on the cell surface, suggesting that the lack of GPI‐APs can also promote complement deposition on the cell surface. These results demonstrate that abnormal complement activation and excessive complement deposition are the basis for thrombotic tendencies in PNH patients. When cells were cocultured with serum from PNH patients receiving C5 antibody therapy, no significant increase in C5b‐9 deposition was observed on cell surfaces even after complement pathway activation. These findings demonstrate that C5 antibodies effectively block C5b formation and inhibit the abnormal and excessive activation of the terminal complement, representing one mechanism through which they reduce TE incidence.

A DVT model confirmed a thrombotic tendency in PNH mice, with more severe phenotypes in Piga/Muc4‐DKO mice. Increased deposition of C5b‐9 was observed in the local thrombotic tissue of Piga/Muc4‐DKO mice, suggesting that *MUC4* mutation may promote the deposition of C5b‐9, which could be a mechanism for promoting thrombus formation in PNH patients. After knockdown of MUC4 expression in K562 KO cells and coculture with PNH‐TES, the deposition of C5b‐9 on the cell surface further increased. The above experiments proved that *MUC4* mutation promotes the development of a thrombotic phenotype in PNH patients by increasing the deposition of terminal complement C5b‐9.

Clinically, PNH patients with high clone size or high disease activity have a high thrombosis risk.^44^, ^45^ Complement inhibitors are indispensable for PNH thrombosis management and are the only proven TE‐preventive intervention.^5^ Primary thromboprophylaxis is not recommended for complement inhibitor users,^46^ whereas anticoagulant–complement inhibitor combinations are preferred for secondary prevention.^47^, ^48^ However, in China, <30% of PNH patients receive complement inhibitor therapy (due to drug cost/accessibility),^49^ making primary thromboprophylaxis for this population a critical clinical priority. For such patients, antiplatelet agents (e.g., aspirin and clopidogrel) are not recommended—despite in vitro evidence of abnormal platelet activation/aggregation in PNH—given their failure to reduce thrombosis risk and increase bleeding risk.^50^ The use of warfarin for TE prevention remains controversial,^51^, ^52^ and direct oral anticoagulants may be effective for PNH‐related TE prevention but lack sufficient research support.^30^, ^53^, ^54^ Guidelines recommend heparins for TE treatment and prevention in PNH,^54^, ^55^ with LMWH being more widely used for primary thromboprophylaxis due to its favourable bleeding risk profile;^56^ this informed our choice of LMWH for prophylactic anticoagulation in mouse experiments, although importantly, anticoagulants alone are insufficient in many cases.^32^, ^53^ Unfortunately, owing to the lack of mouse complement inhibitors, we could not evaluate the impact of complement inhibition on TE incidence in Piga/Muc4‐DKO mice in vivo; however, we demonstrated that prophylactic LMWH significantly alleviated their thrombotic phenotype – with only slight prolongation of BT and CT in mice and no acute haemorrhage or bleeding‐related mortality observed. On this basis, we hypothesise that for PNH patients with concurrent MUC4 mutations, early complement inhibitor initiation (where available) or prophylactic LMWH (for those unable to access complement inhibitors) could be potential strategies for primary TE prevention – although bleeding risk post‐anticoagulation must be closely monitored. Notably, our study has several limitations: a small PNH patient sample size, potential unmeasured confounding factors (e.g., unrecorded comorbidities affecting coagulation) and a lack of prospective clinical follow‐up data. Thus, these potential strategies still warrant further study and prospective validation.

In summary, we propose a possible mechanism for the occurrence of TEs in PNH patients: the abnormal activation and excessive deposition of complement caused by *PIG‐A* mutation are the basis for the thrombotic tendency in PNH patients, and the mutation/down‐regulation of MUC4 further promotes the deposition of abnormally activated terminal complement C5b‐9 on the cell surface, leading to a severe thrombotic phenotype. The specific mechanism by which *MUC4* mutation promotes C5b‐9 deposition and the effects of different *MUC4* mutation sites on the thrombotic phenotype of patients warrant further investigation. In our current clinical practice, we are striving to expand mutation screening to more patients, aiming to identify PNH patients with high‐risk thrombotic factors for the primary prevention of thrombosis. This approach seeks to reduce the incidence of TEs and improve long‐term patient outcomes, but we hope that future data will provide more robust evidence to support our findings.

## AUTHOR CONTRIBUTIONS

Guang Sheng Ling and Rong Fu conceived the study and designed the experiments. Yingying Chen, Mengting Che and Chaomeng Wang performed the experiments. Yingying Chen and Weixin Chen analysed the data. Yingying Chen and Hui Liu wrote the paper. and Chunyan Liu, Guang Sheng Ling and Rong Fu revised the manuscript. All the authors read and approved the final manuscript. Guang Sheng Ling and Rong Fu contributed equally to the study.

## ETHICS STATEMENT

All research involving human blood samples in this study was conducted in accordance with the Declaration of Helsinki and approved by the Ethics Committee of Tianjin Medical University General Hospital (Approval No.: IRB2022‐KY‐029). Prior to participation, all the study subjects or their family representatives were fully informed of the research details and signed a written informed consent form. The animal experiments in this study were approved by the Laboratory Animal Welfare and Ethics Committee of Tianjin Medical University General Hospital (Approval No.: IRB2022‐DWFL‐039) and were conducted in accordance with the Guide for the Care and Use of Laboratory Animals.

## CONFLICT OF INTEREST STATEMENT

The authors declare no conflicts of interest.

## Supporting information



Supporting Information

Supporting Information

## Data Availability

The data that support the findings of this study are available in Bioproject at https://dataview.ncbi.nlm.nih.gov/object/PRJNA1118348?reviewer=ostn0k77cs2sal4qik24087nkp.
