# Supplementary material for: MUC4 mutations promote a thrombotic phenotype in patients with paroxysmal nocturnal haemoglobinuria by increasing the deposition of terminal complement
Source: Clin Transl Med. 2026 Jan 5;16(1):e70567. doi: 10.1002/ctm2.70567 (PMC12771652; doi:10.1002/ctm2.70567)
Supplement: Supplementary file 1 — Supporting Information [file CTM2-16-e70567-s001.docx]

**Supplemental Material-1**

5 Supplemental tables

6 Supplemental figures

Table S1. Clinical characteristics of patients/healthy controls

| Clinical feature | Measurement | | | Reference range |
| --- | --- | --- | --- | --- |
|  | TE (N=12) | HC (N=12) | PNH (N=25) |  |
| Sex,n(%) |  |  |  |  |
| Male | 6 (50.0) | 7 (58.3) | 15 (60.0) |  |
| Female | 6 (50.0) | 5 (41.7) | 10 (40.0) |  |
| Age(years),median age(range) | 53 (32-73) | 38 (24-46) | 36(17-68) |  |
| Blood examination |  |  |  |  |
| RBC(×10^12^/L) | 4.56±0.51 | 4.97±0.51 | 2.19±0.47 | 4.30 ~ 5.80 |
| WBC(×10^9^/L) | 7.74±1.71 | 6.29±1.32 | 4.47±1.86 | 3.50 ~ 9.50 |
| HGB（g/L） | 139.40±13.90 | 148.30±16.37 | 68.72±15.23 | 130 ~ 175 |
| PLT(×10^9^/L) | 260.80±67.62 | 242.30±43.54 | 105.90±75.94 | 125 ~ 350 |
| Ret% | - | - | 7.20±4.20 | 0.50 ~ 1.5 |
| LDH (U/L) | 177.90±30.07 | 187.40±34.15 | 1214.00±771.10 | 120 ~ 250 |
| TBIL (μmol/L) | 10.08±4.52 | 9.62±2.47 | 29.50±15.46 | ≤26 |
| IBIL (μmol/L) | 7.23±3.42 | 6.49±1.42 | 19.26±10.26 | 19 ~ 26 |
| PT (s) | 10.54±0.74 | 11.08±0.74 | 11.73±1.26 | 9.5 ~ 15.0 |
| PT-INR | 0.96±0.07 | 1.01±0.07 | 1.07±0.12 | 0.80 ~ 1.50 |
| APTT (s) | 31.92±2.95 | 30.83±3.97 | 26.32±3.06 | 20.0 ~ 40.0 |
| TT (s) | 17.11±2.00 | 19.37±1.45 | 22.55±7.77 | 13.0 ~ 25.0 |
| FIB (g/L) | 3.49±0.63 | 3.15±0.47 | 3.03±1.26 | 1.80 ~ 4.0 |
| D-dimer (ng/mL) | 574.10±411.10 | 327.50±80.35 | 1678.00±2242.00 | 0 ~ 500 |
| FHB (mg/L) | - | - | 173.40±176.60 | 0 ~ 40 |
| Haptoglobin (g/L) | - | - | 0.20±0.33 | 0.5 ~ 2 |
| CD59^-^Erythrocyte (%) | - | - | 40.66±28.23 | ＜1 |
| CD59^-^Granulocyte (%) | - | - | 77.59±15.99 | ＜1 |
| CD14-FLAER^-^ (%) | - | - | 78.05±16.21 | ＜1 |
| CD24-FLAER^-^ (%) | - | - | 80.06±18.14 | ＜1 |

(RBC: red blood cell, WBC: white blood cell, HGB: hemoglobin, PLT: platelet, Ret: reticulocyte, HP: haptoglobin, LDH: lactic dehydrogenase; TBIL: total bilirubin; IBIL: indirect bilirubin; PT: prothrombin time; PT-INR: prothrombin time - international normalized ratio; APTT: activated partial thromboplastin time; TT: thrombin time; FHB: free hemoglobin; HP: haptoglobin; FLAER: Fluorescently Labeled Aerolysin)

Table S2. Specific primers of *MUC4* and *GAPDH*

| Name | Sequence (5'to3') |
| --- | --- |
| *MUC4* | Forward, CACAACTCTCCCAAAAACAACA |
|  | Reverse, TGTGATGTGTGTCTATCCAGCA |
| *GAPDH* | Forward, CTGGGCTACACTGAGCACC |
|  | Reverse, AAGTGGTCGTTGAGGGCAATG |

Table S3. siRNA sequences of *MUC4*

| Name | Sequence (5'to3') |
| --- | --- |
| siRNA-MUC4 | Forward, GGAUUAGAAAGAUGACAAAdTdT |
|  | Reverse, UUUGUCAUCUUUCUAAUCCdTdT |

Table S4. Clinical characteristics of MUC4 WT and MT patients in the PNH-TE and PNH non-TE groups

| Clinical feature | PNH-TE | | | PNH non-TE | |
| --- | --- | --- | --- | --- | --- |
|  | MUC4 WT (N=4) | MUC4 MT(N=8) | MUC4 WT (N=12) | | MUC4 MT(N=1) |
| Sex,n(%) |  |  |  | |  |
| Male | 3 (75.0) | 6 (75.0) | 6 (50.0) | | 0 (0) |
| Female | 1 (25.0) | 2 (25.0) | 6 (50.0) | | 1 (100) |
| Median age(range) (years) | 47.5(36-67) | 52.5 (25-68) | 33 (17-67) | | 23 |
| Blood examination |  |  |  | |  |
| RBC(×10^12^/L) | 2.47±0.20 | 2.02±0.35 | 2.06±0.48 | | 3.28 |
| WBC(×10^9^/L) | 5.15±1.58 | 4.45±1.64 | 4.39±2.18 | | 3.0 |
| HGB(g/L） | 84.0±13.44 | 66.13±7.08 | 61.67±10.13 | | 113.0 |
| PLT(×10^9^/L) | 81.0±41.0 | 129.0±87.52 | 102.8±80.1 | | 58.0 |
| Ret% | 6.41±2.77 | 9.75±4.55 | 6.0±4.0 | | 4.4 |
| LDH (U/L) | 998.5±723.0 | 1737.0±850.3 | 961.1±633.7 | | 925.0 |
| TBIL (μmol/L) | 28.08±13.59 | 40.34±17.73 | 22.23±11.13 | | 35.9 |
| IBIL (μmol/L) | 19.55±9.20 | 23.94±12.5 | 15.30±8.18 | | 28.1 |
| PT (s) | 11.55±1.86 | 12.26±1.30 | 11.58±1.0 | | 10.1 |
| PT-INR | 1.01±0.17 | 1.12±0.12 | 1.06±0.09 | | 0.9 |
| APTT (s) | 27.4±5.47 | 26.43±2.04 | 25.88±3.0 | | 26.4 |
| TT (s) | 19.0±1.65 | 22.76±4.68 | 23.88±10.46 | | 19.0 |
| FIB (g/L) | 4.10±0.79 | 2.81±1.35 | 23.88±10.46 | | 3.0 |
| D-dimer (ng/mL) | 1781.0±1179.0 | 3208.0±3415 | 738.1±571.3 | | 313.0 |
| FHB (mg/L) | 175.3±181.6 | 304.0±237.8 | 92.17±54.02 | | 95.0 |
| Haptoglobin (g/L) | 0.21±0.20 | 0.09±0.13 | 0.28±0.45 | | 0 |
| CD59^-^Erythrocyte (%) | 46.0±40.15 | 40.45±20.48 | 40.05±31.74 | | 28.34 |
| Clinical feature | PNH-TE | | | PNH non-TE | |
|  | MUC4 WT (N=4) | MUC4 MT(N=8) | MUC4 WT (N=12) | | MUC4 MT(N=1) |
| CD59^-^Granulocyte (%) | 83.13±11.93 | 85.39±6.55 | 72.0±19.35 | | 60.12 |
| CD14^-^FLAER^-^ (%) | 81.65±11.70 | 86.47±7.73 | 73.16±19.16 | | 55 |
| CD24^-^FLAER^-^ (%) | 83.68±11.22 | 89.54±9.46 | 73.94±22.13 | | 63.16 |
| *PIG-A* mutation, n(%) |  |  |  | |  |
| Yes | 2 (50.0) | 4 (50.0) | 4 (33.3) | | 1 (100.0) |
| No | 2 (50.0) | 4 (50.0) | 8 (66.7) | | 0 (0) |

(RBC: red blood cell, WBC: white blood cell, HGB: hemoglobin, PLT: platelet, Ret: reticulocyte, HP: haptoglobin, LDH: lactic dehydrogenase; TBIL: total bilirubin; IBIL: indirect bilirubin; PT: prothrombin time; PT-INR: prothrombin time - international normalized ratio; APTT: activated partial thromboplastin time; TT: thrombin time; FHB: free hemoglobin; HP: haptoglobin; FLAER: Fluorescently Labeled Aerolysin)

Table S5. Blood routine and coagulation function test results of mice after LMWH/PBS administration

| Indicators | PBS | LMWH 100IU/kg/d | LMWH 300IU/kg/d | *p*-value |
| --- | --- | --- | --- | --- |
| Hb (g/L) | 124.2±6.06 | 124.4±12.30 | 122.8±12.93 | 0.9685 |
| WBC (×10^9^/L) | 3.11±1.29 | 3.33±1.27 | 3.58±0.84 | 0.8179 |
| RBC (×10^12^/L) | 6.23±0.76 | 5.96±0.35 | 6.16±0.55 | 0.7964 |
| PLT (×10^9^/L) | 630.4±138.7 | 595.6±95.97 | 635.4±151.7 | 0.8733 |
| BT (s) | 39±1.87 | 44.4±1.52 | 50±1.58 | ＜0.0001**** |
| CT (s) | 153.8±2.49 | 165.6±4.28 | 207.6±6.27 | ＜0.0001**** |

(Hb: hemoglobin, WBC: white blood cell, RBC: red blood cell, PLT: platelet, BT: bleeding time, CT: clotting time) (**** p＜0.0001)

**Figure S1.** Identification of PNH cell model and PNH mouse model. A. The expression level of CD59 in K562 WT and K562 KO: Blue is the experimental tube, red is the isotype control tube; B. The expression level of GPI-AP (CD24/CD48) in peripheral blood cells of PNH mice (*Pig-a* [Flox/Flox, Vav-iCre], *Pig-a* [Flox/Y, Vav-iCre]) and normal C57BL/6 mice.

**

**Figure S2.** Construction of *Muc4* whole-genome knockout mice and *Pig-a* and *Muc4* double-knockout mice in the hematopoietic system. A. Targeting strategy for the generation of *Muc4* whole-genome knockout mice (Muc4[-/-]); B. PCR identification of the *Muc4* gene deletion in *Muc4* knockout mice (numbers 22 and 23), with a mutant (MT) band of 483 bp and a wild-type (WT) band of 8158 bp; C. Sequencing of the PCR amplification product confirming the 7675 bp deletion in the *Muc4* knockout mice; D. Breeding strategy to construct the hematopoietic tissue-specific *Pig-a* and *Muc4* double knockout mice (Muc4[-/-], Piga[flox/flox], Vav-icre / Muc4[-/-], Piga[flox/Y], Vav-icre); E. Western blot validation of Muc4 and Piga expression levels in the bone marrow cells of *Muc4* whole-genome knockout mice (Muc4-KO), hematopoietic-specific *Piga* knockout mice (Piga-KO), and hematopoietic-specific *Pig-a* and *Muc4* double-knockout mice (Piga/Muc4-DKO).

**Figure S3.** Construction of a mouse model of deep vein thrombosis (DVT) in the lower limb. A. Surgical procedure for constructing the DVT mouse model by ligating the unilateral iliac vein; B. Euthanizing mice at different time points after vein ligation, and using a stereomicroscope or H&E staining to observe thrombus formation. Determine that 24 hours after ligation is the ideal time for model establishment (Created By Biorender).

**Figure S4**. Correlation analysis of MUC4 relative expression with disease indicators of PNH patients. (**p*<0.05, ***p*<0.01, ****p*<0.001，*****p*<0.0001) (HGB: hemoglobin, WBC: white blood cell, RBC: red blood cell, PLT: platelet, Ret: reticulocyte, LDH: lactic dehydrogenase; TBIL: total bilirubin; IBIL: indirect bilirubin; FHB: free hemoglobin; HP: haptoglobin; Gra: granulocyte)

**Figure S5.** Flow cytometry results showing complement C5b-9 deposition on the surface of K562 WT (A) or K562 KO cells (B) after coincubation with serum from PNH patients in PBS, GVBMG, or GVB2^+^ buffer, respectively.

**Figure S6**. LMWH treatment significantly decreases the thrombus length in DVT mouse. A. Measurement of the local thrombus length (10×) in different treatment groups 24 h after DVT modeling; B. Statistical analysis of thrombus length in WT, Muc4-KO and Piga-KO mouse. (**p*<0.05, ***p*<0.01, ****p*<0.001, *****p*<0.0001)
